# Metabolites From Trypanosome-Infected Cattle as Sensitive Biomarkers for Animal Trypanosomosis

**DOI:** 10.3389/fmicb.2022.922760

**Published:** 2022-07-14

**Authors:** Merid N. Getahun, John Ngiela, JohnMark O. Makwatta, Peter Ahuya, Tawich K. Simon, Samuel K. Kamau, Baldwyn Torto, Daniel Masiga

**Affiliations:** ^1^International Centre of Insect Physiology and Ecology, Nairobi, Kenya; ^2^Kabete Veterinary Laboratories, Nairobi, Kenya

**Keywords:** animal trypanosomosis, metabolites, biomarkers, diagnostic, volatile

## Abstract

Trypanosomes are important global livestock and human pathogens of public health importance. Elucidating the chemical mechanisms of trypanosome-relevant host interactions can enhance the design and development of a novel, next-generation trypanosomosis diagnostics. However, it is unknown how trypanosome infection affects livestock volatile odors. Here, we show that *Trypanosoma congolense* and *Trypanosoma vivax* infections induced dihydro-β- ionone and junenol, while abundance of dihydro-α-ionone, phenolics, *p*-cresol, and 3-propylphenol significantly elevated in cow urine. These biomarkers of trypanosome infection are conserved in cow breath and the urine metabolites of naturally infected cows, regardless of population, diet, or environment differences. Furthermore, treating trypanosome-infected cows reduced the levels of these indicators back to the pre-infection levels. Finally, we demonstrated that the potential of some specific biomarkers of phenolic origin may be used to detect active trypanosome infections, including low-level infections that are not detectable by microscopy. The sensitivity and specificity of biomarkers detection are suited for rapid, robust, and non-invasive trypanosomosis diagnosis under field conditions.

## Introduction

Trypanosomes are significant infectious diseases that seriously threaten both animals’ and humans’ health and life (Cayla et al., 2019). Trypanosomes are transmitted mainly by tsetse flies and mechanically by other biting flies, and infect a wide range of livestock and wildlife, thereby causing economic losses by inducing mortality, abortion, and morbidity (Auty et al., 2012, 2015; Shaw et al., 2014). While the prevalence of Human African Trypanosomosis (HAT) has reduced in recent years (Aksoy et al., 2017), animal trypanosomosis including zoonotic once, also known as (nagana) remains one of the most significant infectious disease threats to livestock sector, even beyond tsetse fly infested areas (Jones and Dávila, 2001; Garcia et al., 2016; Getahun et al., 2020a; Kimenyi et al., 2021). A better understanding of livestock–trypanosome interactions is the key to effectively control trypanosomosis. *Trypanosoma congolense* and *Trypanosoma. vivax* are the main causative pathogens of animal trypanosomosis (Coustou et al., 2010; Auty et al., 2015; Morrison et al., 2016; Saini et al., 2017).

During blood feeding by infected tsetse flies and other vectors, trypanosome pathogens move into the skin of mammalian hosts, where they distribute into blood, then to particular organs, and tissues, multiply in number and undergoing morphological changes (Coustou et al., 2010). The coevolutionary arms race between the trypanosome and livestock host for their adaptation by means of natural selection may provide many opportunities to study the adaptation mechanism from molecular and chemical level that has a practical application. A secondary metabolite, which are effectors that can act between cells, across tissues and on the host immune system may be a primary mechanism for host-pathogen adaptation (Yang et al., 2018). For instance, *Trypanosoma brucei* infection altered quantities of indole-pyruvic acid, aromatic ketoacids, 4-hydroxypyruvic acid, and phenylalanine in mice (McGettrick et al., 2016; Campbell et al., 2019; Cockram et al., 2020; Pays and Nolan, 2021; Fitzgerald et al., 2022). Furthermore, *T. brucei gambiense* infection in humans induced sleep-inducing metabolites, such as 5-hydroxytryptophan (Vincent et al., 2016). However, our understanding of the volatile metabolites induced from the infection with other trypanosomes such as *Trypanosoma congolense* and *T. vivax*; the two most important livestock trypanosomes in relevant hosts (livestock) are limited. Moreover, earlier studies have shown that *T. vivax* is refractory in mice, making experimental work using model animals with this trypanosome difficult.

Unlike the human diseases biomarkers for example dimethyl disulphide and *p*-menth-1-en-8-ol as cholera biomarkers are identified from feces (Garner et al., 2009). Dimethyl trisulphide breast cancer biomarker (Shirasu et al., 2009), acetone, is found in breath as diabetic indicator (Buszewski et al., 2007). However, there has been little progress in identifying volatile biomarkers from livestock associated with infectious diseases that could be used as disease indicators (Moore et al., 2007). By implementing a controlled infection experiment, followed by field samples and drug treatment validation, here we show that trypanosomes rewire the host volatile organic compounds (VOCs) profile. The identified biomarkers can identify nagana infection in cattle, goat, and surra in camel.

## Results

### Prevalence of Trypanosomes in Cattle

**FIGURE 1:**
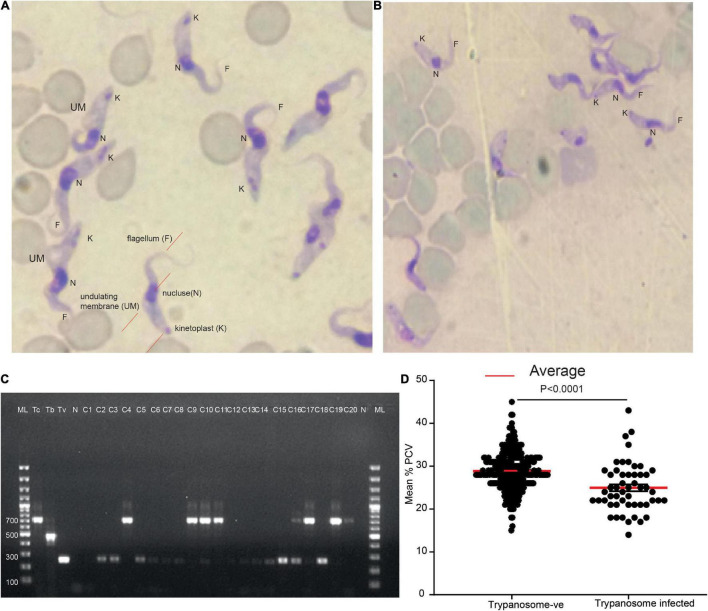
Two major trypanosome species were detected from naturally infected cows. **(A)** Representative light micrographs of Giemsa-stained cow blood sample smears infected with *T. vivax* (look the wavy shape and long flagellum). **(B)** Representative light micrograph of Giemsa-stained cow blood sample smears infected with *T. congolense* showing the less wavy shape and shorter in length flagellum. **(C)** Molecular confirmation of the two trypanosomes. ML 100 bp marker, Tc +ve control for *T. congolense*, Tb, *T. brucei*, and Tv, *T. vivax*, and N, negative control, C1–C20 cows blood sample. **(D)** The mean PCV of cow microscopically trypanosome negative and positive from field samples, *n* = 371.

The presence of trypanosomes in jugular blood obtained from zebu cattle in high tsetse/trypanosome prevalent area of Kenya, Shimba Hills in Coastal Kenya, was investigated. Microscopy analysis of blood samples collected from these animals revealed a trypanosome infection prevalence of 14% (52/371) (Figures 1A,B). *T. congolense* accounted for 63% of total infection (33/52), while *T. vivax* accounted for 37% (19/52); however, this difference was not statistically significant (chi-square, *P* > 0.05). The identity of the microscopically positive samples was further confirmed using PCR by amplifying ITS1 marker (Njiru et al., 2005; Getahun et al., 2020a; Figure 1C). Cows infected with trypanosomes had a low Packed Cell Volume (PCV) (24.98 ± 0.79), a measure of anemia *P* < 0.0001, Mann–Whitney test as compared to microscopically non-infected cows (28.85 ± 0.25) (Figure 1D). Trypanosome positive animals were treated with the trypanocide drug diminazene aceturate (Giordani et al., 2016).

### *Trypanosoma congolense* Infection Reduced Packed Cell Volume

**FIGURE 2:**
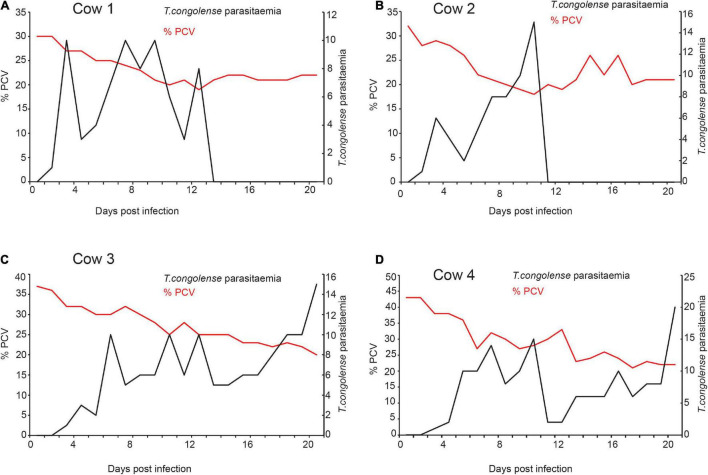
Trypanosome infection caused anemia in infected cows **(A–D)**. The relation between *T. congolense* parasitemia and change in PCV. Right *Y*-axis black dotted graph is the change in parasitemia (number of trypanosomes/ml blood) from pre-infection (day 0) to 20 days post-infection. (>10 trypanosomes per field is equivalent to ∼ > 5 × 10^5^ trypanosomes/ml blood; and 1–10 trypanosome per field is equivalent to ∼10^4^ 5 × 10^5^ trypanosomes/ml blood). Left *Y*-axis the red-dotted line is the measure of Packed Cell Volume (PCV) recorded as a percentage of total blood volume, it measures the level of anemia from pre-infection (day 0) to 20 days post-infection. PCV < 25 considered anemic.

After the trypanosome was isolated from field-infected cow and further multiplied in goats, four cows were subsequently experimentally infected with *T. congolense* and another four cows with *T. vivax*. Within 5 days of infection, cows infected with *T. congolense* showed patent parasitemia (Figure 2). PCV was used as a measure of anemia; PCV significantly reduced within 8 days post-infection, *t* = 2.89, *P* = 0.03 and remained low afterward (Figures 2A–D). Two of the cows were able to clear the parasite after 14–15 days post-infection without treatment (Figures 2A,B). However, the parasites in two cows increased with time (Figures 2C,D), resulting in a substantial decrease in PCV. At day 22 post-infection, the conditions of the two cows deteriorated are (high body temp 40°C, morning anal), anemic, labored breathing, dull, raised hair, weak, and stopped eating. All the four cows were then treated with diminazene aceturate (Veriben), at a dose of 7 mg/kg (Giordani et al., 2016). After treatment, the PCV started to improve, resulting in the survival of all four cows.

### Signature Scent of *Trypanosoma congolense* Infection

**FIGURE 3:**
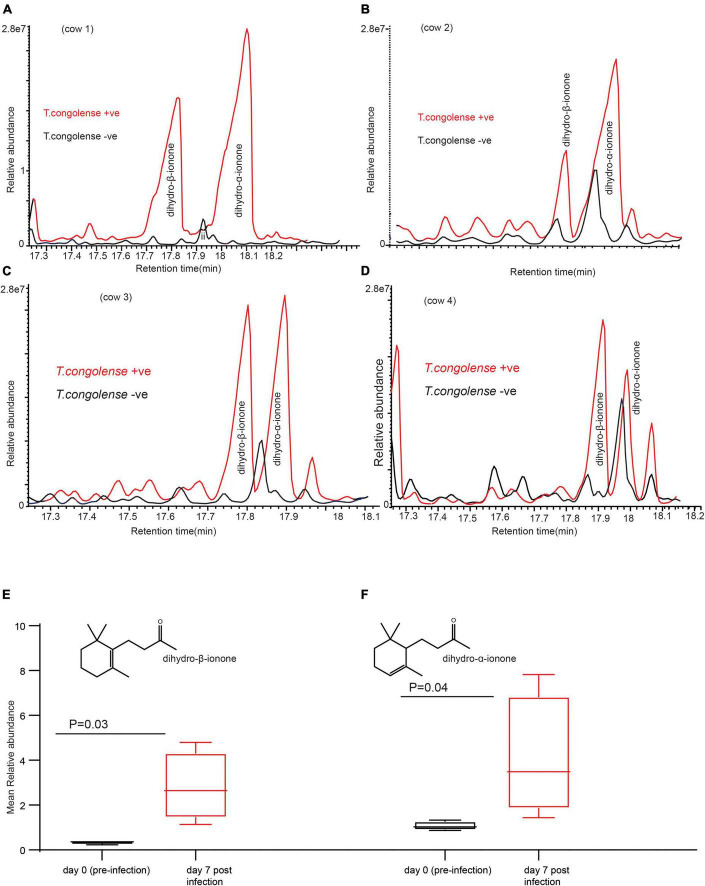
*Trypanosoma congolense* infection induced dihydro-β-ionone in urine metabolites. **(A–D)** GC-MS profiles showing dihydro-β-ionone and dihydro-α-ionone levels in *T. congolense*-uninfected and infected cows, and **(E)** graph showing the significant change in abundance of dihydro-β-ionone due to *T. congolense* infection **(F)**. Graph showing the significant change in a relative abundance of dihydro-α-ionone due to *T. congolense* infection. *Y*-axis in **(A–D)** is the area of the peak, which reflects the amount of a given compound. The size and area of the component peak are proportional to the amount of the component reaching the detector. *X*-axis in **(A–D)** is retention time in minute, which is a measure of the time taken for a compound to pass through a chromatography column. It is calculated as the time from injection to detection.

Next, we wanted to identify which volatile compounds were enriched or induced as a result of trypanosome infection. To address this question, we used coupled gas chromatography mass spectroscopy (GC-MS) to analyze the volatile organic compounds in urine samples. Compared to the urine profile of the same cow before infection (healthy state), the profile of volatile metabolites in urine of cows infected with *T. congolense* showed a significant change in two key components identified as dihydro-β-ionone was induced and dihydro-α-ionone concentration was increased (Figures 3A–F). The mean relative abundance of dihydro-β-ionone was significantly higher in the infected cow urine than that of the healthy state (the same cow prior infection), paired *t*-test *t* = 3.25, *P* = 0.03, and *n* = 4. Likewise, relative abundance of the dihydro-α-ionone level was significantly higher in the infected cow urine than in the healthy state, paired *t*-test *t* = 3.46, *P* = 0.04, and *n* = 4. Our result shows *T. congolense* infection induced dihydro-β-ionone production and increased dihydro-α-ionone levels in cow urine metabolites suggesting that these two metabolites are predictive biomarkers for animal trypanosomosis.

### *Trypanosoma congolense* Modified Metabolites Are Conserved in *Trypanosoma vivax* Infection

**FIGURE 4:**
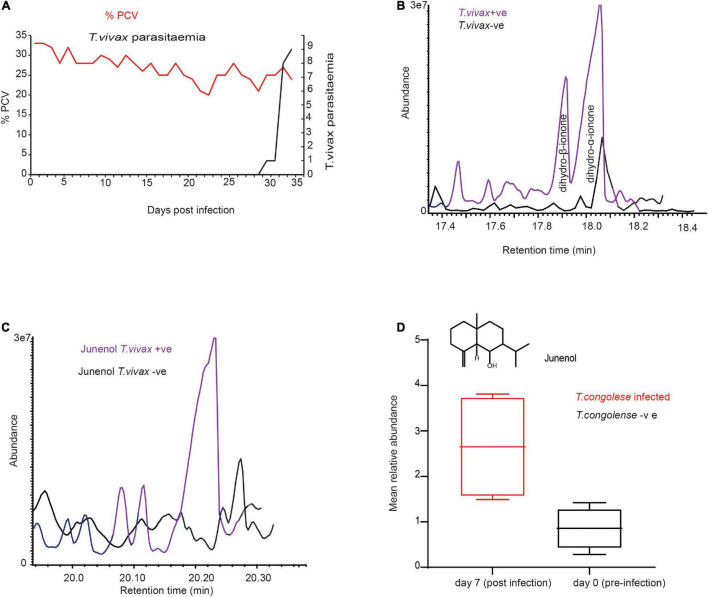
*Trypanosoma vivax* infection induced similar metabolites. **(A)** Parasitemia and PCV change over time in *T. vivax*-infected cow. Left *Y*-axis the red dot is the measure of PCV it signifies the level of anemia from pre-infection (day 0) to 33 days post-infection, PCV < 25 considered anemic. Right *Y*-axis, black-dotted graph is the change in parasitemia (number of parasites/ml blood).>10 trypanosomes per field is equivalent to ∼ > 5 × 10^5^ trypanosomes/ml blood; and 1–10 trypanosome per field is equivalent to ∼10^4^ – 5 × 10^5^ trypanosomes/ml blood. **(B)** GC-MS profile showing dihydro-β-ionone induction and dihydro-α-ionone enhancement due to *T. vivax* infection, **(C)** GC-MS profile showing the induction of junenol due to *T. vivax* infection. **(D)** The mean signal intensity of junenol in healthy and *T. congolense*-infected cow urine, *n* = 4, paired *t*-test 3.96, *P* = 0.02.

We next wondered if these trypanosome biomarker metabolites are specific to *T.congolense* infection. We answered this question by experimentally infecting cows with *T.vivax*, and another species of trypansome. *T.vivax* strain was used and exhbited a different feature, for instance *T.vivax* infection was detected at day 33 post-infection (Figure 4A) and *T. vivax* did not develop in three of the cows, with unknown reason, but it could be that these individual cows were trypanotolerant for the strain. However, *T.congolense* induced biomarkers were conserved in the cow infected with *T. vivax*. Dihydro-β-ionone was induced, whereas dihydro-α-ionone concentration was increased (Figure 4B). The other potential biomarker molecule, which was induced due to both trypanosomes infection was junenol (Figures 4C,D). These findings demonstrate that these two different trypansome species manipulate the host metabolites in a similar manner. At day 35 cow showed *T.vivax* infection curatively treated with diminazene aceturate (7 mg/kg).

### Trypanosome’s Infection Increased Phenolic Compounds Abundance in Cow Urine

**FIGURE 5:**
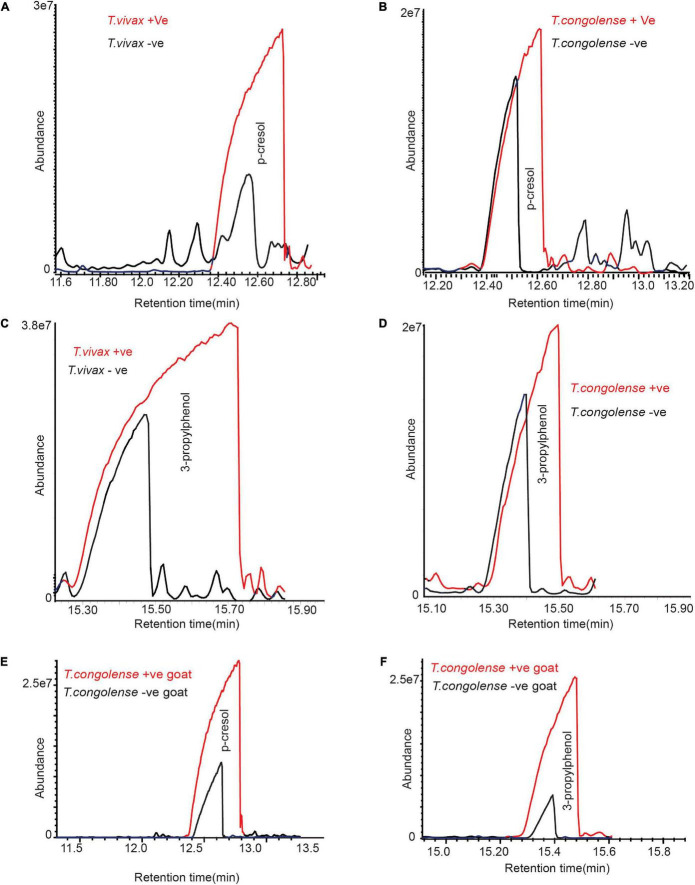
Trypanosome infection increased phenolic compounds concentration in cow urine. **(A–D)** Representative GC-MS profile showing the significant increase of phenolic compounds due to *T. congolense* and *T. vivax* infection. **(E,F)** Representative GC-MS profile showing the significant increase of phenolic compounds due to *T. congolense* in goat urine. Panel **(A)** is for *T. congolense* and **(B)** is for *T. vivax* infection. Due to the variation of metabolites modification between individual livestock, the graphs are not presented at the same scale.

Beside induced metabolites, we looked at metabolites that are present in healthy cow urine, but their concentration increased significantly. Both *T. congolense* and *T. vivax* infection significantly increased the abundance of phenolic compounds in infected cows. The amount of *p*-cresol and 3-propylphenol in cow urine significantly increased with *T. congolense* and *T. vivax* infection (Figures 5A–D). For instance, the relative abundance of *p*-cresol increased on average by fourfold, paired *t*-test *t* = 3.06 and *P* = 0.03. Similarly, 3-propylphenol levels increased by 1.5 times in the urine of trypanosome-infected cows, paired *t*-test *t* = 4.85 and *P* = 0.01. Trypanosome infection, on the other hand, had no effect on the relative abundance phenolic component 4-ethylphenol in cow urine odor, paired *t*-test *t* = 1.12 and *P* = 0.33. In the urine sample of goats infected with *T. congolense*, the amounts of the total phenolic compounds increased significantly, paired *t*-test *t* = 3.03 and *P* = 0.03 (Figures 5E–F).

### Trypanosomes Indicative Metabolites Are Conserved in Naturaly Infected Cow Urine

**FIGURE 6:**
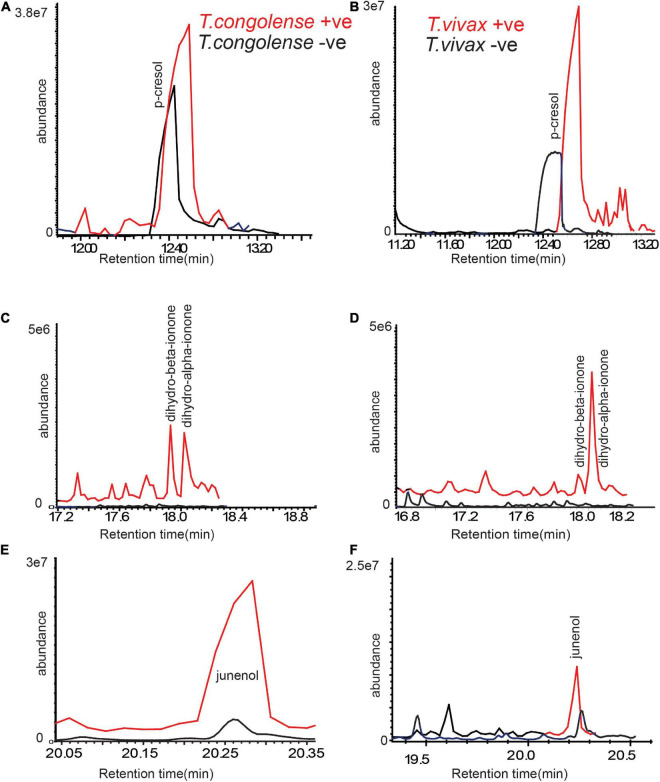
Trypanosome-induced and modified metabolites are conserved in naturally infected cattle urine. **(A,C,E)** Representative GC-MS profiles of trypanosomosis biomarkers in urine of naturally *T. congolense*, (red line) infected cow as compared to healthy cow (black line) and **(B,D,F)** for *T. vivax*. Given the variation of metabolites modification between individual livestock, the graphs are not presented at the same scale.

To compare the consistency of the identified metabolites as trypanosomosis biomarkers, we employed separate field data sets independent of cow populations, environment, and diet. The volatile metabolic profile of urine from cows naturally infected with *T. congolense* and *T. vivax*, (positive by microscopy and PCR) sampled from Shimba Hills, Coastal Kenya, was then examined. We found a high levels of *p*-cresol and 3-propylphenol in the urine of cows naturally infected with *T. congolense* and *T. vivax*. Dihydro-α-ionone, dihydro- β-ionone, and junenol were also induced in naturally infected cow urine (Figures 6A–F). These results demonstrate that trypanosome-indicative biomarkers are conserved in naturally infected cow urine.

### Trypanosomosis Biomarker Molecules Are Conserved in Cow Breath Odors

**FIGURE 7:**
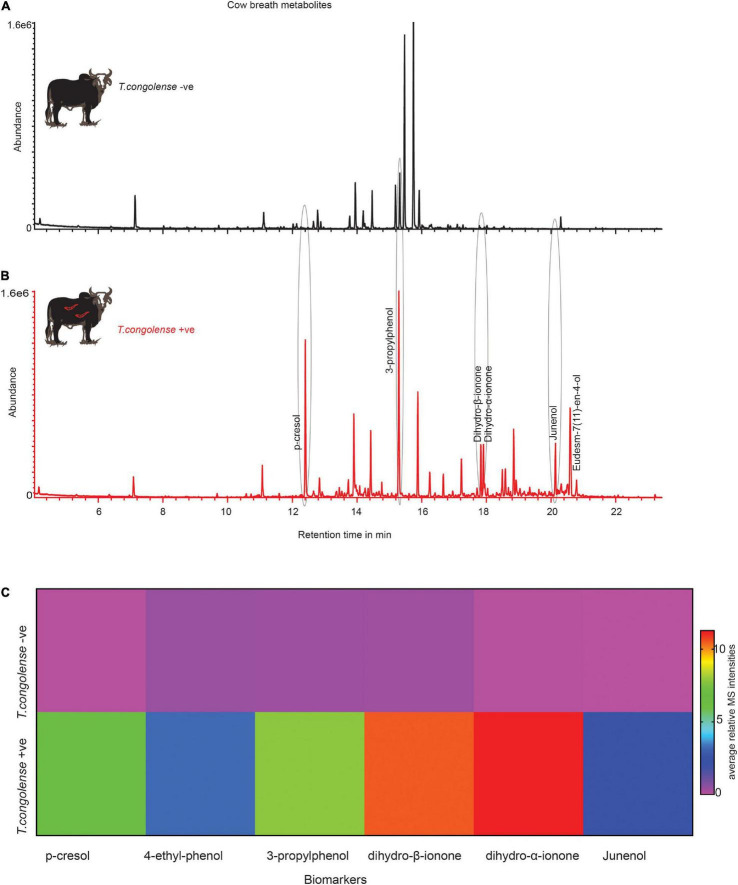
*Trypanosoma congolense* infection induced similar metabolites in cow breath. **(A)** Representative GC-MS trace showing the breath odor components of healthy cow. **(B)** *T. congolense*-infected cow breath. **(C)** The heat map based on the mean relative MS intensities of breath metabolites induced due to *T. congolense* infection, *n* = 4.

We next asked whether these metabolites indicative of trypanosomosis are conserved in other metabolic products, particularly in breath. Following that, we compared the odor profile of cow breath. We discovered that the breath odor profiles of cows infected with *T. congolense* markedly differed from those of healthy cows. The six potential biomarkers identified in urine were induced in cow breath odors as a result of trypanosome infection (Figures 7A,B). Phenolic compounds, ionone, and junenol were not detected in the breath of healthy cows; however, these metabolites were dominant in the breath of cows infected with *T. congolense* (Figures 7A,B). Thus, biomarker molecules including phenolic compounds were conserved in cow breath due to *T. congolense* infection. However, breath odor profiles of healthy cows were dominated by other volatiles including cymene isomers and hydrocarbons, which were suppressed after trypanosome infection. Breath odor components that were induced due to *T. congolense* infection are represented in a heatmap based on their relative percent area in GC-MS intensities (red color for high intensity and purple for low intensity) (Figure 7C).

### Treatment of Experimentally Infected Cow Restores Biomarkers to Pre-infection Levels

**FIGURE 8:**
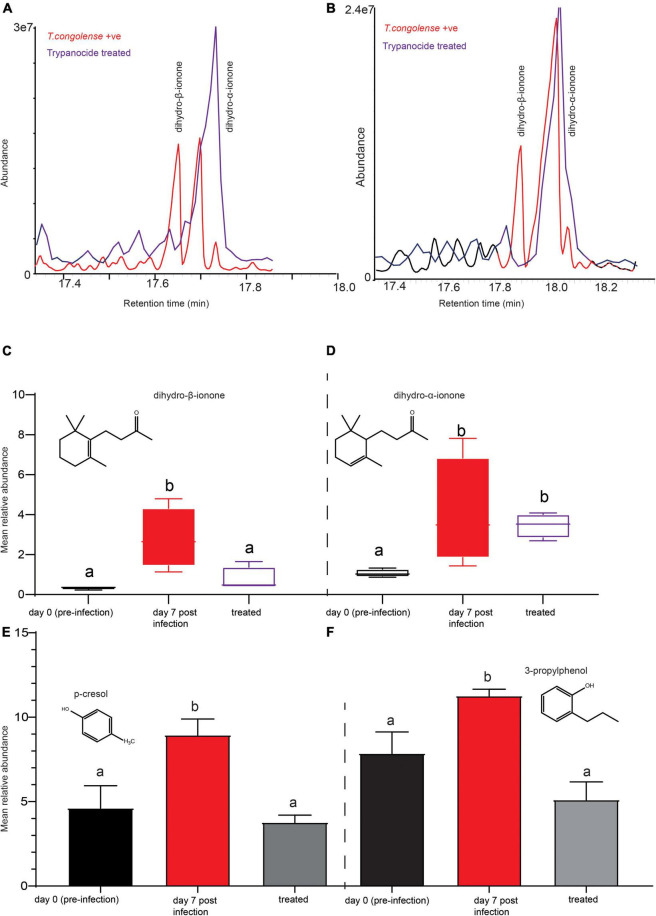
Trypanocides treatment restored the metabolites back to normal. **(A,B)** Representaive GC-MS profiles showing key biomarker levels in trypanosome-infected (red) and after trypanocide treatment (purple). **(C)** The mean relative abundance of dihydro-β-ionone **(D)** dihydro-α-ionone at pre-infection, day 7 post-infection and after treatment. **(E)** The mean relative intensity of *p*-cresol and **(F)** 3-propylphenol at pre-infection, day 7 post-infection and after treatment. In **(C–F)**, bars followed by different letters are statistically significant. Error bars represent standard error of the mean.

We next questioned whether treating the diseased cow would restore the volatile metabolites profile back to normal. One month after trypanocide drug treatment, fresh urine was collected from the same cows and metabolites were analyzed in the same way. The intensity of dihydro-β-ionone has been returned to pre-infection levels (Figures 8A–C). This demonstrates that this biomarker was highly correlated with the trypanosome infection, as evidenced by the reduced emission after trypanocide treatment. However, dihydro-α-ionone was found to be more abundant as compared to dihydro-β-ionone in healthy cow urine, but significantly increased as trypanosome infection remained high (Figures 8A,B,D). Furthermore, the intensity of phenolics (*p*-cresol, and 3-propylphenol) restored to that of uninfected cows’ levels; ANOVA, *F* = 7.168, *P* = 0.01 for *p*-cresol and, ANOVA, *F* = 9.854, *P* = 0.005 for 3-propylphenol (Figures 8E,F).

### The Biomarkers Identified Trypanosome-Infected Cows Correctly With High Sensitivity

**FIGURE 9:**
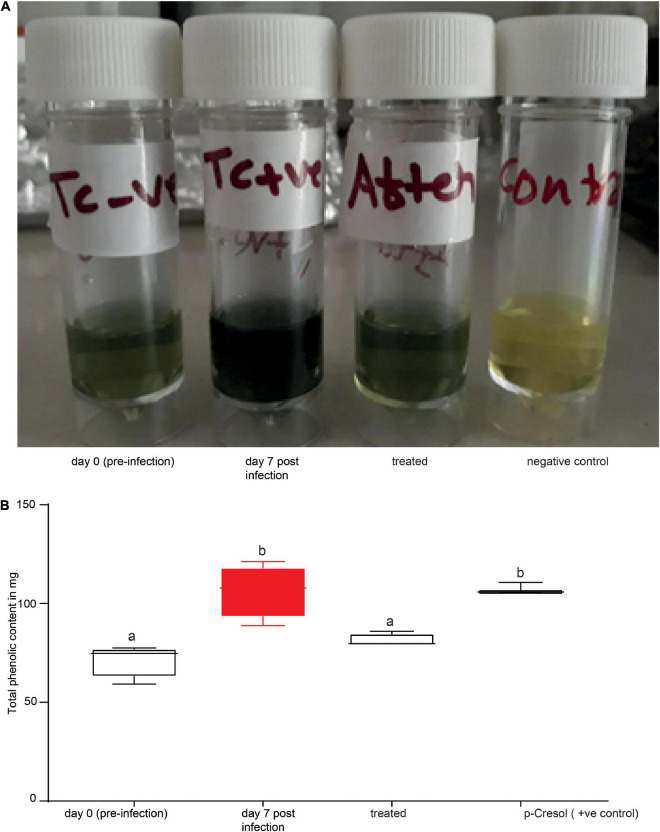
Phenolic biomarkers can be used to diagnose animal trypanosomosis. **(A)** Shows the urine of the same cow before (day 0 pre-infection), during infection (day 7 post-infection) and after treatment. **(B)** Spectrophotometer data quantifying the total phenolic content in the urine of healthy, infected, treated cow (verbine) and 500 ng/μl *p*-cresol (positive control) as standard. Bars followed by different letters are statistically different, ANOVA followed by Tukeys *Post hoc* test, *n* = 4. Error bars represent standard error of the mean. Folin–Ciocalteu reagent upon reacting with phenolics, it produces a blue color which absorbs at 760 nm and the intensity increases linearly with the concentration of phenolics in the sample.

Finally, we inquired as to whether our biomarkers could be used to diagnose cows suffering from trypanosomosis. The Folin–Ciocalteu reagent was used to target phenolic biomarkers. Phenols in biological sample extracts react with Folin–Ciocalteu reagent to form a blue complex that can be quantified by visible-light spectrophotometry. Here, using 1 ml of 10% (1 ml urine diluted in 9 ml distilled water) urine solution of trypanosome-infected and healthy cows, we proved that the biomarkers can clearly and accurately identify infected and uninfected cows with 100% accuracy (Figure 9A). Treament of infected cows with trypanocides (verbine) clearly confirmed that our biomarkers identified an active infections accurately (Figures 9A,B). We next used a spectrophotometer (Beckman DU 650, Beckman Coulter, Inc., Brea, CA, United States) to quantify the total phenolic contents in the healthy, infected, and treated cow urine using the Folin Ciocalteu reagent at 760 nm (Figure 9B). Trypanosome infection increased the total phenolic content in the urine (Figures 9A,B) (ANOVA *F* = 16.91, *P* = 0.0002), which accounted for the color change from green to blue. The color of the urine sample was restored to that of a healthy cow after treatment with trypanocide, confirming the sensitivity of our approach for detecting an active trypanosome infection.

We also wondered if the blue color change in urine caused by the Folin Ciocalteu reagent was linked to high concentration of phenolic biomarkers. To answer this question, we qauntified the concentration of the primary phenolic compound, *p*-cresol, in the headspace of infected and healthy cow urine.We created a calibration curve with *m*-cresol injections of 10, 50, 100, and 1000 ng/μl. The concentration of *p*-Cresol in infected cows’ urine was 500 ± 21 ng/μl, whereas it was 190 ± 60.95 ng/μl in healthy cows’ urine. Dilutions of 1 ml of 500 ng/μl and 200 ng/μl of *p*-cresol samples with 9 ml distilled water, in the same way as the natural urine sample. Our findings clearly demonstrated a color change in 500 ng/μl solution, giving the expected blue color (Figure 9B). On the other hand, a 200 ng/μl *p*-Cresol solution did not change color. We also tested the other biomarkers, ionones, and 10% of 500 ng/μl but they did not result in a color change (data not shown).

To rule out the possibility that the blue color change was caused by other metabolites, we used Urisys 1100 Analyzer (Roche) to measure certain constituents in urine which are significant of renal, urinary, and metabolic disorders using urine test strip. Both infected and healthy cow urine had normal glucose, urobilinogen, and negative for bilirubin and no blood in the urine. There was no difference in pH and specific gravid. Furthermore, when tested using 0.2 N Folin Ciocalteu reagent followed by spectrophotometer, liquid-liquid (urine: hexane 1:1 ratio) extraction reduced the total phenolics content by 74% demonstrating the role of volatile phenolics for the blue color. Protein levels in infected cow urine significantly increased, which could be due to tyrosine, a precursor of phenolics (Tisdall, 1920). We obtained a similar color change when 2 ml of 10% tyrosine was tested using the Folin Ciocalteu reagent and further screened on the UV-Visible spectrophotometer; however, the same concentration of the benzenoid amino acids tryptophan, and phenylalanine failed to give the expected blue color, demonstrating the specificity of the test to phenolics and its precursor. However, to detect a blue color using tyrosine, the concentration has to double when compared to urine or *p*-cresol. The Folin Ciocalteu reagent does not detect protein directly, so the color change may not be a direct reflection of the protein change. Our results confirm that the change in color was due to the phenolic biomarkers and to a lesser extent to phenolic precursors, mainly tyrosine.

### Biomarkers Identified Animal Trypanosomosis From Field Samples With High Accuracy and Sensitivity

**FIGURE 10:**
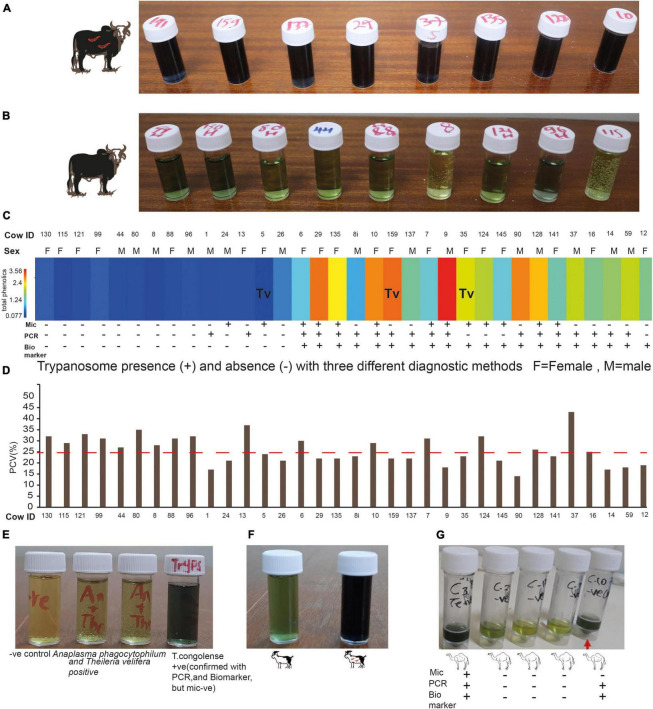
Field validation of the biomarker-based diagnostic. **(A)** Representative cow urine samples from trypanosome positive cows after treatment with diagnostic reagent (see the dark blue color). **(B)** Representative cow urine samples from trypanosome negative cow samples (observe the green color). **(C)** Heat map of the total phenolic contents (unnormalized) from independent field samples both infected and uninfected cows, *n* = 34. (mic, microscopy; PCR, biomarker). Tv, *T. vivax* and the rest infected with *T. congolense*. **(D)** PCV of the 34 cows presented in **(C)**, the dashed line shows the threshold PCV value to be considered anemic (PCV < 25). **(E)** Specificity test. **(F)** Trypanosomosis diagnosis in goat. **(G)** Camel trypanosomosis (surra) diagnosis.

The value of identified biomarkers in our controlled experiment is reinforced when cross-validated with heterogeneous independent samples. To validate our novel animal trypanosomosis diagnostic method developed using controlled experimental results, we sampled independent blood from cows in the field at Shimba Hills, coastal Kenya in November 2020. Blood samples were screened for trypanosomes using microscopy which were then validated by PCR. From trypanosome postive cattle simultaneously, we collected urine before treatment to validate our novel diagnosis using independent samples. We also collected blood and urine samples from cow with low PCV, <25% anemic cows, an indication of trypanosomosis or other blood pathogen infections. Urine was also collected from 10 healthy cows. We used our novel diagnostic tool to screen 34 independent simultaneous blood and urine samples collected in the field (Figures 10A–C). Out of 34 animals (15 males and 19 females), 10 were healthy (negative for trypanosome by microscopy and PCV > 25%). Thirteen cows were positive for trypanosomes microscopically and eight of them were anemic. Sixteen cattle were anemic, PCV < 25%, but negative for trypanosome microscopically (Figure 10). For confirmation and comparison, we used microscopy, PCR, and our novel biomarker-based diagnostic. The biomarker diagnostic showed a high prediction accuracy and sensitivity per sample and works for both male and female cattle (Figures 10A–C). All three diagnostic approaches had 100% agreement in diagnosing trypanosome infection status in the 18 samples out of 34 samples analyzed. But two samples that were microscopically positive for trypanosome were diagnosed negative both using PCR tests and our biomarkers. In another two samples that were trypanosome positive with PCR, they were found to be negative both microscopically and with the biomarker. Eight samples obtained from cows with low PCV (mean PCV 21 ± 2, as low as 14 CowID 90) were microscopically negative, but PCR and biomarkers confirmed them to be trypanosome positive. One urine sample, from cow 12 with low PCV was found to be negative both microscopically and with PCR but trypanosome was positive with biomarkers (Figure 10C). In another three samples (cow 35, 124, and 145), microscopy and biomarkers showed similar results, trypanosome positive, but negative with PCR (Figure 10C).

### The Specificity of the Novel Diagnostic Tool

We wanted to know how specific is our novel biomarker-based animal trypanosomosis diagnosis in differentiating other livestock diseases. To evaluate its specificity, we challenged our novel biomarker-based nagana diagnostic using urine from five cows from Shimba Hills Coastal Kenya, that were anemic, with average PCV of 21.2 ± 1.1, raised hair, inappetence, and poor body condition. The absence of trypanosomes in these cows was confirmed microscopically, followed by a PCR test. These cows, on the other hand, were discovered to have a mixed infection of *Anaplasma phagocytophilum* (Gene bank accession no MZ443994) that causes anaplasmosis in cattle, and *Theileria velifera* (Gene bank accession number MZ441074) that causes theileriosis in cattle. The urine from these animals did not give a blue color with our diagnostic (Figure 10E), showing our novel biomarkers-based diagnosis was specific for animal trypanosomosis. Our diagnosis also worked for *T.congolense*-infected goats (Figure 10F) and *T.evansi* (surra)-infected camel (Figure 10G).

### Biomarker-Based Animal Trypanosomosis Diagnostic Versus Standard Methods

The test agreement between our novel diagnostic method with the existing golden standard microscopy and PCR methods showed that there was a fair agreement between microscopy and our biomarker-based diagnostic with Kappa coefficient of 0.4, 95% confidence interval from 0.099 to 0.658. However, there was a substantial agreement between PCR and our novel method, with kappa coefficient of 0.64, 95% confidence interval from 0.457 to 0.942. Similarly, the test agreement between microscopy and PCR was fair with kappa coefficient of 0.3, 95% confidence interval from 0.035 to 0.584. High response range from low parasitemia (asymptomatic) to high parasite load were detected with our novel diagnostic demonstrating that it has wider dynamic detection range.

## Discussion

Animal African trypanosomiasis caused by *T. congolense* and *T. vivax* is still one of the most serious livestock illnesses in sub-Saharan Africa, affecting millions of cattle every year (Alsan, 2015; Morrison et al., 2016; Rodrigues et al., 2019; Ngari et al., 2020), our data showed that high trypanosomes prevalence (14%) and infected cattle were considerably anemic, an indication of trypanosomosis severity (Mamoudou et al., 2016), compared to trypanosome-negative cattle, necessitating improved vector control tools, diagnostics, and therapeutics to improve the health and productivity of livestock. Here using epidemiologically important pathogens in relevant livestock host, we demonstrate the potentiality of biomarkers as a model mechanism for animal trypanosomosis diagnostics.

Volatile organic compounds usually reflect the health condition of an individual, therefore, getting an infectious disease often results in a change in VOCs (Shirasu and Touhara, 2011). Hence, investigating changes in metabolites using relevant pathogen–host interaction potentially lead to the discovery of novel biomarkers for infectious diseases diagnostic or therapeutical interventions.

Changes in metabolites are often specific to organs and tissues of an animal (Jones and Dávila, 2001) but it is also known that local responses can spread to other animal tissues or organs, making them accessible for sampling non-invasively. Breath VOCs are endogenous products of physiological/metabolic body processes or products of various microbial pathogens, or they are produced by the host in response to microbial infections, released to the environment via the lungs (Sethi et al., 2013). Similarly, the chemicals in urine are intermediate products or end products of a number of metabolic pathways, and these substances are derived from a variety of chemical classes (Wagenstaller and Buettner, 2013). Here using these two metabolic products (urine and breath) as a representative of the host metabolites, we describe the finding of biomarkers for animal trypanosomosis with evidence that they are conserved between urine and breath metabolites of a cow for certain trypanosome species.

We show that the VOCs metabolites of healthy and infected cows differ markedly, demonstrating that trypanosomes manipulate the cow metabolome during infection. For instance, dihydro-β-ionone and junenol were induced in cow urine during *T. congolense* and *T. vivax* infection, and six biomarkers were induced in cow breath metabolites, which were absent in healthy cow breath odor profile. Furthermore, the relative abundance of phenolic compounds, particularly *p*-cresol and 3-propylphenol, which dominate healthy cow urine metabolites significantly increased several folds in cows infected with both *T. congolense* and *T. vivax*. Similarly, there was a significant increase in the concentration of phenolic compounds in urine of goats infected with *T. congolense*. In one study malaria infection triggers human red blood cells to increase the release of abundant odorants such as CO_2_, aldehydes, and monoterpenes (Emami et al., 2017). In another study, the profiles of VOCs produced by humans and mice infected by *Plasmodium falciparum* and *P. chabaudi* showed upregulation and downregulation in certain compounds (De Boer et al., 2017; De Moraes et al., 2018; Robinson et al., 2018). Among metabolites that showed significant variation between healthy dogs and dogs infected with *Leishmania infantum* include the aldehydes, octanal, nonanal, and decanal (Magalhães-Junior et al., 2019). Urine obtained from camel infected with trypanosomes has been found to smell differently from urine obtained from healthy camels(Hunter, 1986); however, the metabolites accounting for the difference in smell has not been reported.

The similarity of biomarkers induced by trypanosomes in naturally infected cows from field collected urine samples shows that the change is not related to populations, feed, urine water level/hydration or dietary end-products resulting from digestive and excretory processes or microbiota. Since these two groups of animals were kept under different conditions, environment and feeding regime, demonstrating the biomarkers, are unambiguously modified due to trypanosome infection regardless of feeding or population of cattle. The field collected cows were free rangers, while the experimental cows were fed on hay and remained indoors in a fly proof setting. High response range from low parasitemia (asymptomatic) to high parasite load were detected with our novel diagnostic demonstrating it has wider dynamic detection range.

This raises the question of whether the identified biomarkers in our study are specific to trypanosomes infection or they could be a general “scent of infection.” The analysis of metabolites after treatment clearly demonstrated their association with trypanosome infection. Furthermore, urine from cow infected with *Anaplasma phagocytophilum and Theileria velifera* alone or in mixed infection were not identified as positive with our diagnostic technique, showing that our biomarkers are specific for diagnosing animal trypanosomosis. However, their use requires further detail specificity test against several relevant pathogens including babesia. Furthermore, an investigation in cattle infected with *Mycobacterium bovis* which is an infectious disease in cattle identified cyclohexane and pentadine in the breath of infected cattle (Peled et al., 2012). These two odors were not detected in our analysis, showing host–pathogen interaction may have their own signature metabolites or host defense to different pathogen infections may be associated with different metabolites. While the present work does not delve further into other metabolites, such as non-volatiles metabolites that may be modified due to trypanosomes infection, origin of induced metabolites and the biosynthetic pathways of modified metabolites, these would be an interesting avenue for future investigation.

Analysis of infection-related VOCs in exhaled breath and urine potentially enables diagnosis and monitoring of metabolic and pathophysiological processes in a non-invasive way when volatile breath and urine biomarkers can be related to conditions of health or disease. For instance, in humans, volatile biomarkers have been identified from exhaled-breath samples (Miekisch et al., 2004; Boots et al., 2012; Amann et al., 2014), urine (Guernion et al., 2001), and feces (Garner et al., 2007). Furthermore, they have been proven to be useful for diagnosing a broad range of diseases including diabetes (Hu and Galassetti, 2005; Novak et al., 2007), lung disorders (Robroeks et al., 2010; Boots et al., 2012; Mazzone et al., 2015), and infections (Phillips et al., 2007; Chambers et al., 2012). The non-invasive nature of both breath and urine, as well as the conserved emission of biomarkers in both, suggests that breath and urine volatiles as suitable candidates for a rapid diseases diagnosis in livestock. Thus, our trypanosomes biomarkers modified and induced livestock VOCs analysis will open opportunities for diagnosis of various infectious diseases in veterinary medicine, such as an early detection method is important since ∼60% of emerging infectious diseases in humans are of zoonotic origin (Jones et al., 2008).

In conclusion, trypanosome infection causes consistent changes in livestock VOCs emissions, regardless of cattle populations or metabolic products (urine, breath). The novel biomarkers-based diagnosis has good sensitivity (including asymptomatic infection), specificity and it can be used to diagnose three trypanosomes in three different hosts under field conditions. This could lead to the development of a low-cost, easy-to-use, rapid, non-invasive tool that addresses several shortcomings (technicality, cost) of the currently available microscopic and PCR diagnostic methods for animal trypanosomosis Furthermore, our method detects an active infection, overcoming challenges with methods that only measure exposure to pathogens. This demonstrates that these biomarkers can be used in follow-up studies, which may reflect disease progression and in monitoring effectiveness of therapeutic intervention beside active infection detection. Overall, the results of this investigation show that trypanosome-induced metabolites can be used as a powerful alternative diagnostic tool for animal trypanosomosis.

## Materials and Methods

### Ethics Statement

We collected blood, urine, and breath from cow, goat, and injected pathogens in accordance with protocols approved by the International Centre of Insect Physiology and Ecology’s Institutional Animal Care and Use Committee (IACUC) guidelines (approval number: *icipe*-IACUC-10/2018.1). All personnel actively involved in the study completed CITI Program course.

### Trypanosome Species

We aimed to use the currently circulating trypanosomes in livestock for our experiment. Thus, blood samples were obtained from 371 cattle from Shimba Hills, coastal Kenya, where there is high trypanosomes challenge (Saini et al., 2017) in October 2018. Approximately 5–10 mL of blood was drawn from the jugular vein of cattle into vacutainer tubes containing disodium salt of ethylene diamine tetraacetate (EDTA) (Plymouth PLG, Plymouth, United Kingdom) and kept in liquid nitrogen. Before liquid nitrogen storage, an aliquot from each vacutainer tube was transferred into heparinized capillary tubes (75 × 1.5 mm) and spun in a micro-haematocrit centrifuge at 12,000 rpm for 5 min to separate the red and white blood cells and plasma, hence concentrating on the trypanosomes.

We used microscopy to score the trypanosome infection status in cows; the trypanosome species were differentiated based on cell motility and morphology using wet blood film under microscope examination. *Trypanosoma congolense* was recognized by its small size (11–18 μm) as in relation to red blood cell diameter, and its sluggish movement and its invariable attachment to red blood cells. *T. vivax*, on the other hand, was large (18–29 μm) and strikingly apparent by the speed with which it whirred and traversed the microscopic field (Murry, 1978; Bargul et al., 2016; Getahun et al., 2020a).

Packed Cell Volume, an indicator of the animal’s anemic status, was measured using Haematocrit Reader (Hawksley & Sons Limited, Lancing, United Kingdom) and expressed as a percentage of PCV to total blood volume. The buffy coat plasma interface was placed onto a microscope glass slide and examined under a microscope for the presence of moving trypanosomes. Furthermore, thin blood smears were prepared from the samples, fixed with methanol, and stained with 10% Giemsa for microscopic examination. To further confirm our microscopic finding, we employed PCR identification as indicated in Getahun et al. (2020a). For PCR analysis, total genomic DNA was extracted from trypanosomes positive blood samples using DNeasy Blood & Tissue Kits (Cat No./ID: 69504, Qiagen, Hilden, Germany), using DNA-based markers (Masiga et al., 1992; Adams et al., 2006) that enabled differentiation of trypanosome species and their subgroups. We employed PCR targeting the internal transcribed spacer (ITS-1) gene fragment, which is a conserved gene across all African trypanosomes (Desquesnes et al., 2001). Molecular confirmation of trypanosomes was visualized by electrophoresis in 1.2% agarose gel. The diagnostic PCR assays were carried out in 10-μL reaction mixtures containing 5 μL 2 × DreamTaq mix, 3 μL PCR water, 0.5 μL of 10 μM ITS 1 primers (F: 5′-CCGGAAGTTCACCGATATTG-3′; R: 5′-TTGCTGCGTTCTTCAACGAA-3′) (Njiru et al., 2005) and 1 μL DNA template. PCR amplification conditions were programmed as follows: 95°C denaturation step for 1 min, 35 cycles of 95°C for 30 s, 61°C for 30 s, 72°C for 1 min and final extension of 72°C for 10 min.

### Trypanosome Multiplication

From trypanosome-infected cow’s blood, 10 ml blood from *T. congolense*- and *T. vivax*-infected cows, with parasite load 2–3 trypanosomes/view, which is equivalent to ∼10^4^ – 5 × 10^5^ trypanosomes/ml blood, were collected in clean tubes and 2 ml of the blood was injected into a healthy goat, a given goat was injected trypanosome from a single cow for further multiplication and for controlled artificially trypanosome injection experiment. Five goats were injected with *T. congolense* and the other five with *T. vivax*. The trypanosome-injected goats were transported to Nairobi and kept in a fly proof setting at the Directorate of Veterinary Services, Nairobi. Parasite survival and multiplication were checked every 3 days from blood obtained from the ear vein microscopically and body temperature; PCV was also checked whenever blood was sampled.

### Trypanosome Infection Experiment

Local zebu breed (*Bos indicus*) cows were purchased from a tsetse-free area of Central Kenya and examined for any blood parasites (trypanosomes, anaplasma, theileria, etc.), using microscopy followed by PCR. They were all negative for trypanosomes. They were dewormed and kept in a fly proof animal keeping unit at Kabete Veterinary Research Laboratories, Nairobi Kenya. Monoconical traps were placed around the animal enclosure to trap biting flies, and the windows were covered with mesh that does not allow insects. All cows were of the same age (2 years old heifers) and were maintained on hay, wheat bran and allowed to leak salt and to drink water *ad libitum*. The animals were allowed to acclimatize for 70 days before used in further experiments.

### Trypanosome Injection

*Trypanosoma congolense* and *T. vivax* were acquired from a single host and multiplied in a single goat to control or minimize for potential variation between trypanosome strains. *Trypanosoma congolense* (accession number MZ461917) and *T. vivax* (accession number MZ461918) that were isolated from field-infected cow were multiplied in goat. Each trypanosome species was injected into a different goat. A high parasitemia, 20 trypanosomes per field (>6 × 10^7^/ml of blood) was obtained within 10 days. For experimental infection of cows 2 ml of blood with 20 trypanosomes/field (equivalent to ∼ > 5 × 105 trypanosomes/ml blood) were injected through the jugular vein after the cows were restrained. Blood was slowly and gently aspirated into the syringe to prevent air bubbles and then the desired dose, 2 ml of the infected blood was injected. Thereafter, the needle was withdrawn and then a gentle pressure was applied to the puncture site using cotton wool until bleeding ceased. Only one cow out of eight trypanosome-infected cows reacted to trypanosome injection, but it was normalized shortly. The PCV, the parasite load, was checked on day 0 (injection day) then after day 5, followed by every day for 22 days for *T. congolense* and for 33 days post-infection for *T. vivax*.

### Parasitemia and Packed Cell Volume Monitoring in Experimental Cows

To check for parasitemia, the trypanosome-infected cows were restrained, their ear veins were pricked with a clean lancet, and blood was collected using a pair of heparinized micro-haematocrit centrifuge capillary tubes. The capillary tubes were sealed with Cristaseal (Hawksley) and centrifuged immediately in a micro-haematocrit centrifuge for 5 min at 12,000 rpm and the presence of trypanosomes in the blood was determined using the buffy coat (BCT) and wet smear technique. For microscopic examination of the parasite, the capillary tube was cut with a diamond pointed pen 1 mm below the buffy coat to include the uppermost layer of red blood cells and 3 cm above to include the plasma. The cut capillary placed on the slide and covered with a coverslip, then observed carefully by scanning the preparation under a microscope with 40× objective. Furthermore, the PCV which is a measure of anemia quantified was determined using a PCV reader (Hawskley Ltd., United Kingdom). We also measured body temperature via the rectum. Experimental animals and infected animals from field were treated with Diminazene aceturate, by the trade name Veriben with 7 mg/kg dose.

### Odor Collection

Odor collection was done as previously reported (Getahun et al., 2020b) from the healthy, before infection and infected cows, *N* = 4. Approximately, 1 L each of fresh urine was collected from healthy (before infection) and trypanosome-infected cow, day 7 after artificially infected with trypanosomes. This was done after demonstrating that treated cows showed the presence of trypanosomes in their blood (2–4 trypanosomes per view microscopically). However, for *T. vivax*-infected cow, this was done after 33 days of post-infection due to delayed incubation period. Volatiles were collected from the urine for 12 h using a dynamic headspace collection technique on Poropak™ Type Q adsorbent fitted with a portable vacuum pump (all components from Sigma scientific, Micanopy, FL, United States). Clean air was pushed at 2.5 L/min while the vacuum or pull was set at 2 L/min. The trapped odors were eluted with 300 μl GC-MS-grade hexane from Sigma Aldrich, Darmstadt, Germany.

For collection of breath odors, healthy and trypanosome-infected cows were restrained by holding the horn and ear, and then the nose and mouth were enclosed in Teflon bag to concentrate the odors while isolating environmental contaminants. The adsorbent attached to the portable pump pointed directly to the mouth without touching any of the body such as tongue, and the position was alternated between mouth and nose to trap all odors coming out of the animal for a maximum of 30 min, at 15 min intervals (Getahun et al., 2020b). The date of collection was the same as the urine, after the cows showed trypanosome infection. The trapped odors were eluted with 300 μl GC-MS-grade hexane as done for the urine samples. Odor samples were collected in the same cow after trypanocide treatment, followed by parasite clearance confirmation both microscopically and with PCR and drug withdrawal period, after 1 month.

### Chemical Analysis

To characterize and separate the trapped metabolites from urine and breath, we used a gas chromatograph coupled to a mass spectrometer (GC–MS; HP 6890 GC and 5975 MS; Agilent Technologies, Palo Alto, CA, United States) in the electron impact at 70 eV. Helium was used as the carrier gas at an average linear flow rate of 35 cm/s. An autosampler (Agilent Technologies) was used to inject 1 μl of each sample into the GC–MS on a non-polar capillary HP-5 column. Injections of the volatile extracts were conducted in a splitless injector at 220°C. The oven temperature was programmed to 35°C for 5 min and then increased by 10°C/min to a final temperature of 280°C and held at this temperature for 10 min. Mass spectra and retention times of volatiles were compared with their commercial standards where available and library database spectra using the NIST mass spectral program (ver. 2.0), Pherobase^1^, and the NIST web book^2^. We placed emphasis on metabolites that were induced or enhanced when compared to the healthy cow urine and breath. When the potential biomarker compounds were available, we co-injected the standards and compared their spectra and retention times of the predicted compounds to confirm their identities.

### Total Phenolic Content Analysis for Diagnosis

Preliminary analysis showed the presence of phenols in odors of both healthy and trypanosome-infected cows. Since phenolics emission was affected due to trypanosome infection, thus we quantified total phenolics in all urine samples using 0.2 N Folin–Ciocalteu (Sigma Aldrich, Germany) according to previous methods (Singleton et al., 1999; Mokaya et al., 2019). Phenols in biological sample extracts react with specific redox reagents, such as Folin–Ciocalteu reagent to form a blue complex that can be quantified by visible-light spectrophotometry. Briefly, 1 ml of urine from healthy and trypanosome-infected cows was diluted with 9 ml distilled water and then, 1 mL of the resulting solution was mixed with 5 mL of 0.2 N Folin–Ciocalteu reagent. Thereafter, 0.05 g sodium carbonate was added, mixed, and then the mixture was incubated at room temperature for 2–5 min. The absorbance of the reaction mixture was read spectrophotometrically at 760 nm against 0.2 N Folin–Ciocalteu reagent blank. Gallic acid was used as a standard to generate the calibration curve (0–250 μg/ml). The total phenolic content was expressed as mg of gallic acid equivalents. Folin–Ciocalteu reagent upon reacting with phenolics produces a blue color which is absorbed at 760 nm and the intensity increases linearly with the concentration of phenolics in the sample.

### Statistical Analyses

#### Sample Size Calculation

To determine the diversity and prevalence of actively circulating trypanosomes in the livestock in the region, we determined the sample size, animals to be sampled using the formula: $n\,\frac{\ln\,\left(\right)}{\ln\,\left(1-p\right)}$, according to OIE manual for terrestrial animals 2012. Based on our preliminary data, we found cows were infected with three different trypanosomes, *T. brucei* (1%), *T. vivax* (3%), and *T. congolense* (6%). Since *T. brucei* infection was very low, we considered that as reference point to calculate how many cattle to sample. At 95% confidence limit, therefore, α = 0.05, *P* = 0.01, we took the lowest infection rate. Sample size $n\,\frac{\ln\,\left(\right)}{\ln\,\left(1-p\right)}$ therefore *n* = –2.99/–0.01 = 299, indicating that randomly a minimum of 299 cattle to be sampled and examined microscopically.

We employed power analysis (Dell et al., 2002) to determine the number of cows to use to profile the trypanosome-induced metabolites. *N* = (z^2^σ^2^/E^2^), *Z* = 1.96 for 95% confidence, *E* = 5%, to get the Standard Deviation (σ), we analyzed the consistent and major phenolic compounds (*p*-cresol, 4- ethylphenol-, 3-propylphenol) relative abundance from 7 cow urine samples using GC-MS. Our result showed that the variability of these compounds between cows resulted in a standard deviation of 4.6 ∼ 5. Based on our sample size calculation, the number of cows needed to identify specific VOCs attributable to *trypanosome* infection was 3.8; thus, we used four cows for our experiment. The relative abundance of the enhanced/induced VOCs was analyzed using the total relative abundance area of the biomarkers as compared to healthy cow using paired *t*-test. Independent *t*-test was used for independent samples and when the data were not normal, Mann–Whitney test was used. The normality of the data was checked using Leven test. The biomarkers relative concentration and total phenolic contents between healthy, infected, and treated samples were analyzed using ANOVA followed by Tukey’s *Post hoc* test. We used Cohen’s *kappa* coefficient (κ) developed by Landis and Koch (1977), a statistic used to measure agreement between methods for qualitative (categorical) diagnostic methods. Prism (v23, IBM, New York, NY, United States) was used for data analysis.

## Data Availability Statement

The datasets presented in this study can be found in online repositories. The names of the repository/repositories and accession number(s) can be found in the article/supplementary material.

## Ethics Statement

The animal study was reviewed and approved by Animal Care and Use Committee (IACUC) of the International Centre of Insect Physiology and Ecology. Written informed consent was obtained from the owners for the participation of their animals in this study.

## Author Contributions

MG contributed to conception and study design, performed most of the experiments, analyzed and interpreted the data, and wrote the manuscript. JN, PA, JM, TS, and SK contributed to various data generation of the study. DM and BT designed the experiments, revised, and edited the manuscript. All authors read the manuscript.

## Conflict of Interest

The authors declare that the research was conducted in the absence of any commercial or financial relationships that could be construed as a potential conflict of interest.

## Publisher’s Note

All claims expressed in this article are solely those of the authors and do not necessarily represent those of their affiliated organizations, or those of the publisher, the editors and the reviewers. Any product that may be evaluated in this article, or claim that may be made by its manufacturer, is not guaranteed or endorsed by the publisher.

## Footnotes

1
http://www.pherobase.com

2
http://webbook.nist.gov/chemistry
